# Sulphate of Spartein

**Published:** 1888-04

**Authors:** 


### SULPHATE OF SPARTEIN.

A report upon the clinical action of sulphate of spartein, from the clinic of Professor Nothnagel, in Vienna, and based upon eight cases of valvular disease of the heart, four of emphysema, two of pleuritis, and one of catarrhal icterus, appeared in a recent number of the *Medicinisch-Chirurgisches Centralblatt.* From the observations made in the cases mentioned the following conclusions are drawn:

1. Sulphate of spartein is an active cardiac stimulant in small doses. The contractions were more efficient, the pulse fuller, the arterial tension increased, and the frequency of the pulse usually diminished a few beats.

2. The action occurs in from three-quarters of an hour to one hour after administration, often continues longer than twenty-four hours, and can be increased by a repetition of the dose during this time. After a number of days of its employment a cessation of administration is necessary, as it then again acts more strongly. It may be employed for periods longer than a week without ill effects.

3. The disturbance in the rythm of the contractions of the heart will occur again only in a few cases. In the more severe cases of heart-lesions the smaller contractions become indeed more marked, but the force of the stronger is not correspondingly increased.

4. The frequency of respiration is sometimes increased and sometimes diminished, after the administration of the remedy.

5. The diuretic effect often corresponds with the increased force of the contractions of the heart.

6. A secondary slight narcotic effect frequently occurs in the form of increased calm and slumber.

7. With the small doses of one to four milligrammes (1-60 to 1-15 grain), symptoms of intoxication, dizziness, headache, palpitation, and nausea, only seldom occurred, were slight, disappeared quickly, and did not re-appear if the dose was repeated. Spartein can properly displace the infusion of digitalis, but its action appears to increase too rapidly and not to remain at the maximum long enough for it to combat the severe disturbances of compensation, and one can not obtain, through repeated doses of it, such permanent increase in cardiac power as can be obtained by the use of digitalis.

It, however, possesses a great advantage in precision of dosage.

Its therapeutic indications are as follows:

1. In valvular lesions in the stage of disturbance of compensation, when the pulse is moderately full and strong.

2. In valvular lesions without marked disturbance of compensation, as a regulating and quieting remedy.

3. In insufficiency of the heart-muscle without valvular leisons.

4. In pericarditis.

5. As a duretic it is inferior to other medicines.

6. As an adjuvant in connection with digitalis.

